# Weed Roots Facilitate the Spread of *Rosellinia necatrix*, the Causal Agent of White Root Rot

**DOI:** 10.1264/jsme2.ME19013

**Published:** 2019-06-20

**Authors:** Hayato Shiragane, Toshiyuki Usami, Masahiro Shishido

**Affiliations:** 1 Graduate School of Horticulture, Chiba University 648 Matsudo, Matsudo-shi, Chiba 271–8510 Japan

**Keywords:** white root rot, *Rosellinia necatrix*, rescue grass, *Bromus catharticus*, weeds

## Abstract

*Rosellinia necatrix* causes white root rot in various plants, including the Japanese pear. PCR assays using specific primers for *R. necatrix* detected the fungus on the roots of nine weed species from infested pear orchards. The soil inoculation experiment revealed that the spread of *R. necatrix* was similar between weed-mowed and non-weed-mowed treatments under field conditions. The spread of *R. necatrix* was also observed when rescue grass (*Bromus catharticus*) was grown in planter boxes under greenhouse conditions, but was limited without the grass, suggesting that some weeds facilitate the spread of *R. necatrix* in soil.

White root rot is a severe root disease that causes serious economic losses in crop production, particularly in the fruit industry. The causal agent, *Rosellinia necatrix* Prillieax, is an ascomycete that has various host plants ranging from ornamental herbs to fruit and forest trees. This fungus may survive and persist in soil for a long time though survival adaptations, including perithecia or pseudo-sclerotia (4, 7).

Weeds are common on the floors of fruit orchards, and their roots remain alive for some time even after mowing. Weeds may serve as reservoirs to alternative hosts for pathogens and their vectors (13). For example, common lamb’s quarters (*Chenopodium album*) is susceptible to infections of the alfalfa isolate of *Verticillium albo-atrum*; this weed may maintain fungal inocula (2). Frederick *et al.* (3) reported that *V. dahliae* isolates infected 16 weedy hosts in the Pacific Northwest and suggested that weeds, depending on the species, grown during and between potato crop rotations increased the fungal population. Another soilborne fungal pathogen, *Rhizoctonia solani*, survives on many weed species (1). Owing to its various host plants, we assumed that *R. necatrix* also colonizes the roots of some weeds in fruit orchards. Takemoto *et al.* (11) reported that some wild plant species were colonized by this fungus and, thus, potentially serve as inoculum reservoirs of white root rot; however, they mostly examined woody plants with two herbaceous species in forest floors.

In the present study, we initially identified some weed roots in orchards of the Japanese pear (*Pyrus pyrifolia*) that were colonized by *R. necatrix* without exhibiting symptoms of the disease. We then investigated the effects of weeds on the spread of the fungal pathogen using rescue grass (*Bromus catharticus*). We hypothesized that the colonization of *R. necatrix* in weed roots may facilitate fungal spread in soil.

To investigate whether any weed root was colonized with *R. necatrix*, two Japanese pear orchards, known for infestations of white root rot for more than two decades (9), were selected for the sampling of weed plants. The orchards were located in the Center for Environment, Health and Field Sciences, Chiba University, in Kashiwa-shi, Japan (35°53′38 N, 139°56′49 E) with areas of 689 and 673 m^2^.

Twenty weed species, at least one sample per species, were collected from the orchard floors between May and September 2014. The roots of each weed sample were thoroughly washed with tap water and dried under room conditions for 4 d. The total DNA of each root sample was extracted using MagExtractor (Toyobo, Osaka, Japan) in accordance with the manufacturer’s instructions.

Nested PCR was performed for every DNA sample as described previously by Shishido *et al.* (10). Initial PCR amplified the fungal ITS-rDNA region using the primer pair ITS1 and ITS4 (12), with the following cycling conditions: initial denaturation at 95°C for 10 min; 35 cycles of denaturation at 95°C for 15 s, annealing at 60°C for 30 s, and an extension at 72°C for 30 s; and a final extension at 72°C for 7 min, using GeneAmp PCR System 2700 (Applied Biosystems, Thermo Fisher Scientific, Tokyo, Japan). The reaction mixture consisted of BIO*Taq* (0.5 U), Ampdirect (10 μL, Shimadzu, Kyoto, Japan), 0.5 μM of the ITS1 and ITS4 primers, and 2 μL of extracted DNA as a template in a final volume of 20 μL. Since the weeds used in the present study were grown in volcanic ash soil (Andosol), which strongly inhibits DNA extraction and subsequent PCR (10), we applied nested PCR assays along with a gene amplification reagent (Ampdirect) to neutralize PCR inhibitors.

In second PCR, primers specific for *R. necatrix*: R2 (forward, 5′-CAA AAC CCA TGT GAA CAT ACC A-3′) and R5 (reverse, 5′-CAA TGC TAA ACA GAG TTT CGT G-3′), were used under the same cycling conditions as those for initial PCR, but without the Ampdirect reagent. The primer pair R2 and R5 was originally developed by Schena *et al.* (8). Amplicons were visually detected at the expected size of 133 bp using agarose gel electrophoresis after staining with 5% ethidium bromide.

Among the 20 weed species collected from the Japanese pear orchards, *R. necatrix* DNA was detected in nine species, *i.e. B. catharticus*, *Setaria viridis*, *Poa annua*, *Zoysia japonica*, *Digitaria ciliaris*, *Crassocephalum crepidioides*, *Lamium purpureum*, *Arenaria serpyllifolia*, and *Vicia sativa* subsp. *nigra*, in at least one root sample (Table 1 and Fig. S1). Some bands of PCR amplicons specific to *R. necatrix* were weak and this may have been due to root washing, which may have resulted in the removal of fungal mycelia. Alternatively, soil still adhering to the root surface may have inhibited DNA extraction and subsequent PCR. However, none of these plants wilted or developed lesions on the root surface, and all appeared to be healthy. Therefore, these weeds were colonized or infected by the fungus without symptoms. We were unable to visually observe the mycelial strands of *R. necatrix* on roots that were mostly fine and whitish. Since *R. necatrix* has not been reported as pathogenic to these plants, the relationship between this fungus and each host plant may be symbiotic; however, it may be commensalism, not mutualism, wherein weed roots provide *R. necatrix* niches. These ecological relationships need to be verified by further investigations of the fitness status of each organism.

Takemoto *et al.* (11) inoculated two *R. necatrix* isolates into 10 non-host species: eight woody shrub and two herbaceous plants that were common in forest floors in Japan. They found that although the mortality of plants varied, all plant species tested were susceptible, having rotting lesions on the inner bark and cortex. Therefore, the benign symptom observed in the present study in weed species by *R. necatrix* colonization was not consistent with these findings. Various fungi, including plant pathogens, have the ability to colonize weed roots and utilize them as nutrient sources regardless of the ecological status with their host plants (5). Although it is uncommon to find weeds diseased with white root rot, various plant pathogens change their trophic states between pathogenic, parasitic, and symbiotic (6). Therefore, it was not surprising that these nine weed species allowed *R. necatrix* to colonize their roots, providing its unknown niche in orchard soil.

Due to the detection of the white root rot fungus on some weed roots, we hypothesized that weed roots assist in the spread of *R. necatrix*. To confirm our hypothesis, we conducted a field experiment using concrete frames at Chiba University Experiment Farm located at Matsudo-shi, Japan (35°46′36 N, 139°54′39 E). The concrete frames were circular with an inner diameter of 0.8 m and were filled with loam soil (Andosols; 58.0% sand, 35.2% silt, and 6.8% clay). Some of the soil properties were as follows: bulk density, 0.97 kg L^−1^; pH, 6.2; CEC, 210 mmol kg^−1^; total C, 19 g kg^−1^; total N, 1.8 g kg^−1^; available P, 41 mg kg^−1^; exchangeable K, 0.64 g kg^−1^; exchangeable Ca, 2.5 g kg^−1^; exchangeable Mg, 0.43 g kg^−1^.

Four concrete frames had been fallowed for three years, and weeds grew naturally in these frames. The weed species grown in these concrete frames were *B. catharticus*, *Cerastium glomeratum*, *C. crepidioides*, *Cynodon dactylon*, *Cyperus microiria*, *D. ciliaris*, *Equisetum arvense*, and *S. viridis*. All weeds were mowed in two concrete frames, while none were removed from the other two frames. Prior to the inoculation experiment, no *R. necatrix* was detected in the soil of frames upon nested PCR.

A 10-cm-long dried Japanese pear twig, 1 cm in diameter, was autoclaved and aseptically inoculated with *R. necatrix* K64 (MAFF 625120; GenBank Culture Collection of Ministry of Agriculture, Forestry and Fisheries in Japan) in an Erlenmeyer flask, followed by an incubation at 25°C for two months. The pear twig, completely colonized with the fungus, was inserted into the center of the concrete frame as the inoculum source. One week after twig insertion, up to 20-cm-deep soil samples were collected using a 20-cm-long plastic tube at points 3, 10, 20, and 30 cm from the twig, with four replicates at right angles to each other. Four replicated soil samples of the same distance from the inoculum twig were pooled and thoroughly mixed. Total DNA was extracted from 0.5 g of each soil mixture using ISOIL for bead beating (Nippon Gene, Tokyo, Japan). Nested PCR was performed to detect *R. necatrix* as described above. Soil sample collection and nested PCR assays were conducted every two weeks for seven weeks after twig insertion.

The mycelia of *R. necatrix* appeared to spread via weed roots irrespective of whether the shoots were mowed in the concrete frames. Two weeks after setting the inoculation experiment, fungal DNA was detected in soil 20 cm from the Japanese pear twig colonized with *R. necatrix* or the inoculum source, and the area of infestation then gradually expanded with time (Table 2 and Fig. S2). Fungal mycelia reached the edge of the concrete frames or 30 cm from the inoculum source in a similar time regardless of mowing at the third week. Although *R. necatrix* may have spread through the residues of weed roots, the result of this experiment suggested that weed roots facilitated the spread of fungal mycelia even after being mowed in Japanese pear orchards.

To confirm the results of the concrete frame experiment, we conducted another inoculation experiment with artificially growing weed plants in planter boxes under greenhouse conditions. Plastic 13-L planter boxes that were 55 cm long, 18 cm wide, and 18 cm deep were filled with a soil-based medium consisting of 5-mm mesh-sieved field soil and vermiculite mixed in a ratio of 1:1. The source of the loam was the Chiba University Experimental Farm, pasteurized before use.

Rescue grass was used in this experiment because the weed most commonly accommodated *R. necatrix* in the Japanese pear orchards and produced abundant seeds with high germination rates. Seeds were sown at 3-cm intervals in two lines 8 cm apart. The non-weed control received no seeds. Inoculum sources of Japanese pear twigs colonized with *R. necatrix* K64 were similarly prepared and cut to a length of 5 cm. An inoculum twig was buried to a depth of 5 cm in the soil medium and 3 cm from the edge of the planter box simultaneously with seed sowing. The non-inoculation control box received only the grass seeds without the inoculum twig. Each treatment was replicated in three planter boxes. Planter boxes were placed on a bench in a greenhouse in which the temperature ranged between 22 and 38°C. Plants were watered every two d without fertilizer application.

Three months after sowing, soil samples were collected from the points 3, 25, and 50 cm from the inoculum twig. Soil DNA was extracted using ISOIL for bead beating (Nippon gene), and nested PCR to detect *R. necatrix* DNA was performed as described before. Twenty rescue grass samples were randomly harvested from *R. necatrix*-inoculated and non-inoculated planter boxes. Shoots and roots were separated, and their biomasses were assessed after drying at 70°C for 3 d. The means of these variables were statistically tested by the Student’s *t*-test. The inoculation experiment using rescue grass in planter boxes was repeated to ensure reproducibility.

*R. necatrix* DNA was detected from soil samples 3, 25, and 50 cm from the inoculum source in all planter boxes containing rescue grass after three months of sowing of the grass, accompanied by the fungal inoculation (Fig. S3). Since the planter box was 50 cm long, *R. necatrix* appeared to have spread throughout the planter box with rescue grass via the roots at this time. In contrast, in the planter boxes of the nonweed control, *R. necatrix* DNA was only detected in soil collected 3 cm from the inoculum source, and not from the soil sample 25 or 50 cm from the fungal source, suggesting that the fungus extended its mycelia not more than half of the distance of the soil medium with the grass roots. Furthermore, since no significant difference (*P*>0.05) was observed in the biomasses of the shoot and root or in the tiller numbers of rescue grass between the inoculated and non-inoculated treatments (Table 3), the relationship between *R. necatrix* and rescue grass did not appear to be parasitic. *R. necatrix* also developed mycelial strand-like structures on the surface of rescue grass roots (Fig. S4), observed under a dissecting microscope (Olympus SZ2-ILST; Tokyo, Japan) when the grass was grown similarly to the planter box experiment. However, since experimental conditions were semi-compulsory with an artificial inoculation in the pasteurized soil, further experimentation under field or natural conditions is needed in order to clarify whether weed roots play the role of an inoculum reservoir of *R. necatrix*.

In conclusion, the present study showed that *R. necatrix* was accommodated in the roots of nine weed species out of the 20 species collected from two Japanese orchards infested with the fungus. Moreover, two inoculation tests under field and greenhouse conditions revealed that some weed roots, including rescue grass, facilitate the spread of this fungus. Since no root lesions, leaf discoloration, or wilting of infected weeds were detected, *R. necatrix* was not pathogenic to these weeds. Furthermore, there were no significant differences in biomasses between fungal-inoculated and non-inoculated rescue grass, and, thus, some weeds may be able to supply nutrients for the mycelial growth of *R. necatrix* in their rhizosphere.

## SUPPLEMENTARY MATERIAL



## Figures and Tables

**Table 1:** *Rosellinia necatrix* colonization on roots of weed species growing in Japanese pear orchards.

Scientific name	Common English name	Detection of *R. necatrix* by PCR assays^a^	Lanes in Fig. S1
*Bromus catharticus*	rescue grass	+	A9, A10, A11, B13, B14
*Setaria viridis*	green foxtail	+	B15
*Cynodon dactylon*	bermuda grass	−	A5
*Poa annua*	annual bluegras	+	A7, A8, B2, B3
*Zoysia japonica*	Korean lawngrass	+	A2, C2
*Digitaria ciliaris*	southern crabgrass	+	B10, B11, C14
*Cyperus microiria*	Asian flatsedge	−	C3
*Cyperus rotundus*	purple nutsedge	−	A6, B4
*Crassocephalum crepidioides*	ebolo, fireweed	+	C5
*Lamium purpureum*	purple deadnettle	+	A3, A4
*Rumex obtusifolius*	broadleaf dock	−	B5, C4
*Commelina communis*	Asiatic dayflower	−	C7, C8
*Equisetum arvense*	field horsetail	−	B6, C9
*Solanum nigrum*	black nightshade	−	C10
*Cerastium glomeratum*	sticky chickweed	−	C11
*Stellaria media*	common chickweed	−	C13
*Arenaria serpyllifolia*	thymeleaf sandwort	+	B12, C6
*Amaranthus viridis*	slender amaranth	−	C15
*Vicia sativa* subsp. *nigra*	narrowleaf vetch	+	B7, B8, B9
*Trifolium repens*	white clover	−	C12

a Nested polymerase chain reaction assays were conducted using the primer pairs of ITS1/ITS4 followed by R2 and R5 as described by Shishido *et al.* (10).

**Table 2:** Spread of *Rosellinia necatrix* inoculated in concrete frames with or without mowing weeds

Weed treatment	Distance from the inoculum (cm)	Time after inoculation of *R. necatrix* source (weeks)*			

		1	3	5	7
Not mowed	3	−, −	+, +	+, +	+, +
	10	−, −	+, +	+, +	+, +
	20	−, −	+, +	−, +	+, +
	30	−, −	−, −	−, +	+, +

Mowed	3	−, −	+, +	+, +	+, +
	10	−, −	−, +	+, +	+, +
	20	−, −	−, +	−, +	+, +
	30	−, −	−, −	−, −	+, +

* The spread of *R. necatrix* was assessed using fungal-specific polymerase chain reaction assays with the detection of fungal DNA in soil distant from the inoculum source in concrete frames under field conditions. The results of agarose gel electrophoreses were presented in Fig. S2; +: detected, and −: not detected.

**Table 3:** Shoot and root biomasses and tiller numbers of rescue grass (*Bromus catharticus*) with or without an inoculation of *Rosellinia necatrix*

Treatment	Root dry weight (g hill^−1^)	Shoot dry weight (g hill^−1^)	Number of tillers hill^−1^
*R. necatrix* inoculated	0.187±0.008^1)^	1.281±0.075^a^	2.67±0.13
Non-inoculated control	0.183±0.010	1.302±0.074	2.65±0.16
Student’s *t*-test	n.s. (*P*<0.75)	n.s. (*P*<0.85)	n.s. (*P*<0.94)

a Mean±S.E. (*n*=20)
