# Supplementary material for: Weed Roots Facilitate the Spread of Rosellinia necatrix, the Causal Agent of White Root Rot
Source: Microbes Environ. 2019 Jun 20;34(3):340–3. doi: 10.1264/jsme2.ME19013 (PMC6759340; doi:10.1264/jsme2.ME19013)
Supplement: Supplementary file 1 [file 34_340_s1.pdf]

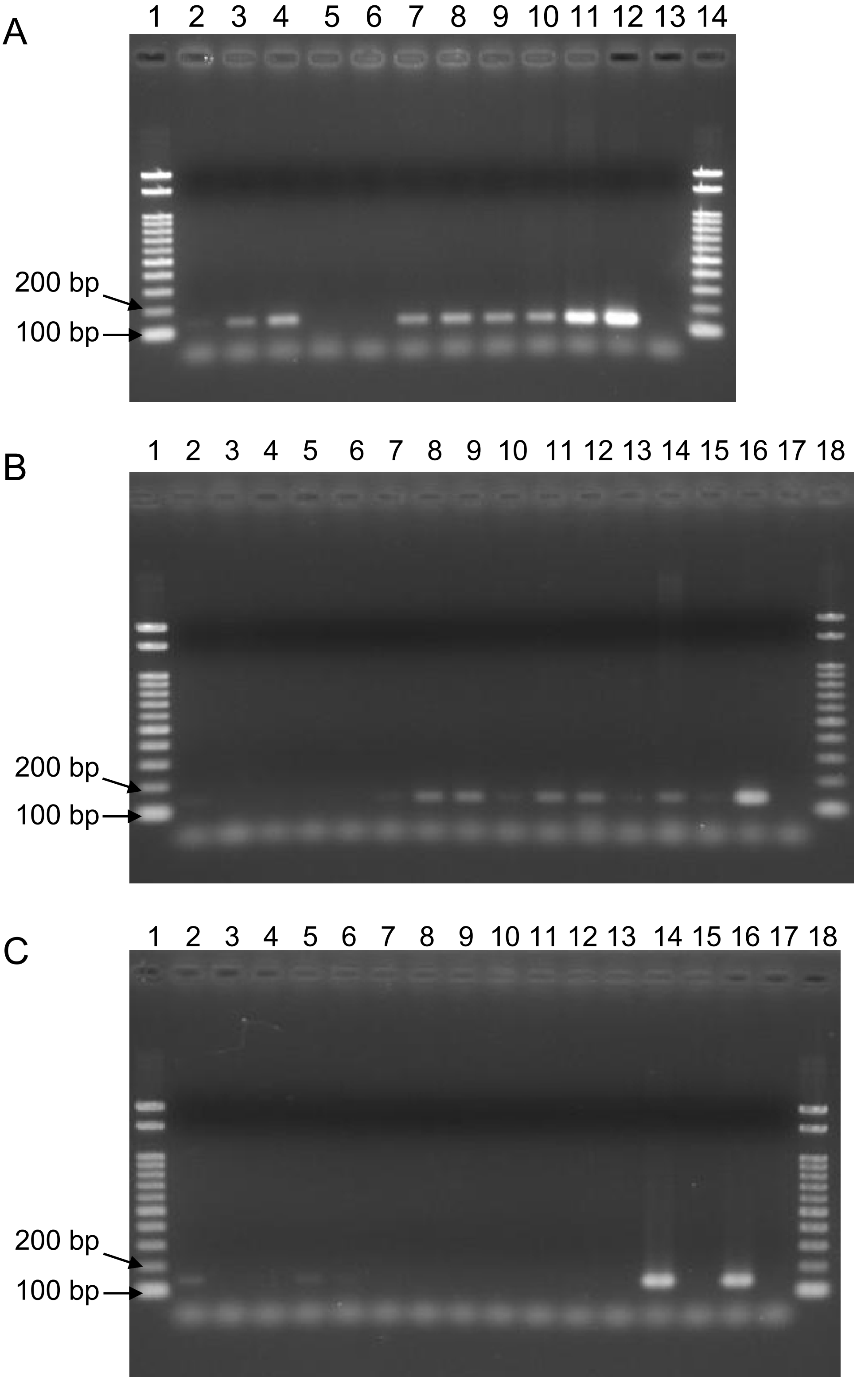

Fig. S1. Agarose gel electrophoreses showing specific bands amplified via nested PCR using DNA templates extracted from weed roots grown in Japanese pear orchards. Lanes A1, A14, B1, B18, C1, and C18 contain the molecular size markers (Gene ladder 100, Nippoin gene Co.). Lanes A12, B16, and C16 contain positive controls (*Rosellinia necatrix* DNA). Lanes A13, B17, and C17 contain negative controls (no DNA templates). DNA templates of other lanes (weed species) are indicated in Table 1.

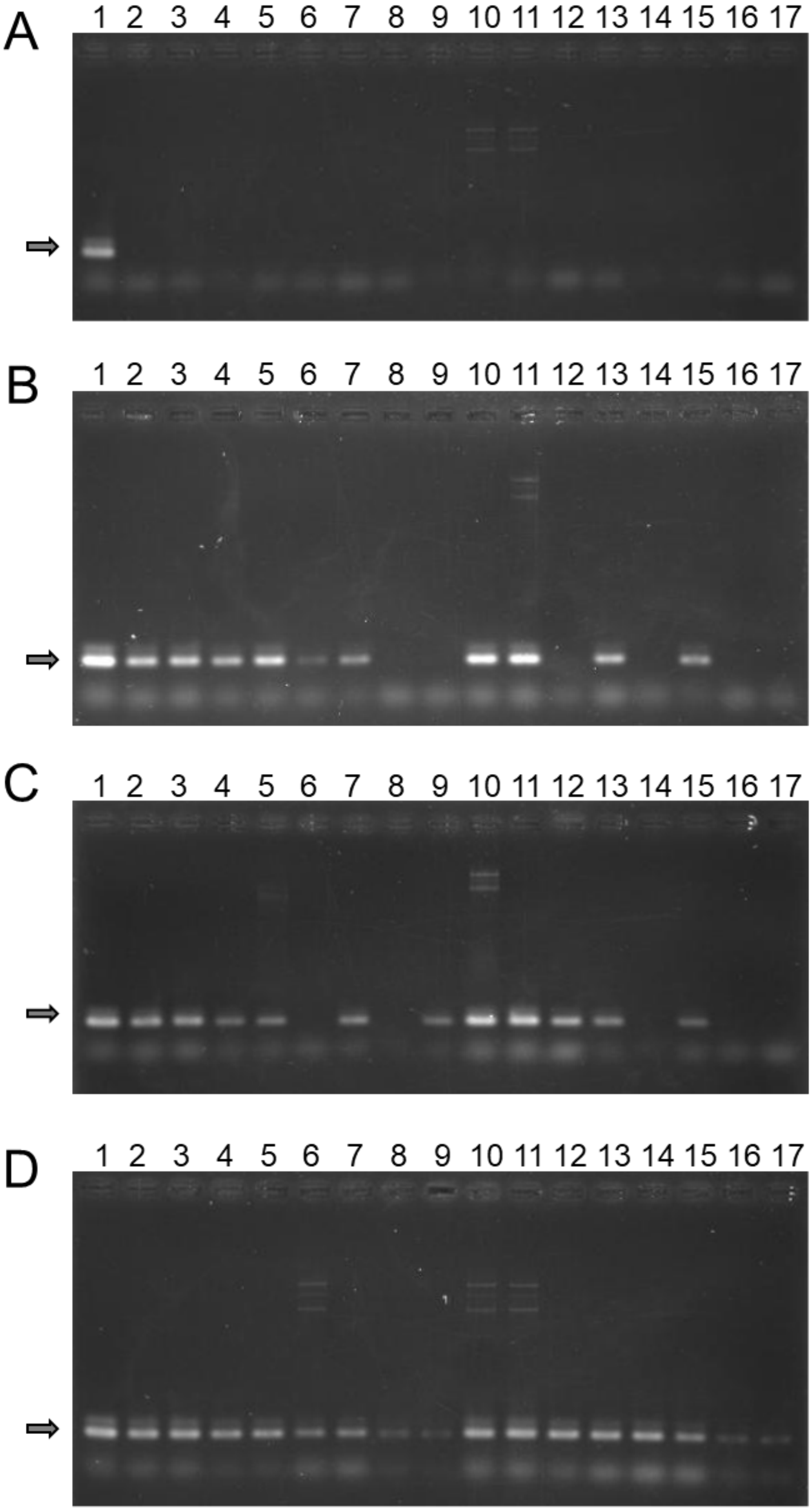

Fig. S2. Agarose gel electrophoreses showing specific band of *Roseliinia necatrix* (arrows) amplified via nested PCR using DNA templates extracted from the soil of concrete frames. Lane 1: positive control (*R. necatrix* DNA). Lanes 2, 3, 10, and 11: 3 cm from the inoculum, Lanes 4, 5, 12, 13: 10 cm from the inoculum, Lanes 6, 7, 14, and 15: 20 cm from the inoculum, and Lanes 8, 9, 16, and 17: 30 cm from the inoculum. Lanes 2 through 9: weeds were not mowed, whereas Lanes 10 through 17: weeds were mowed. A, B, C, and D were the samples collected 1, 3, 5, and 7 weeks after the inoculation of *R. necatrix*, respectively. These results were presented in Table 2.

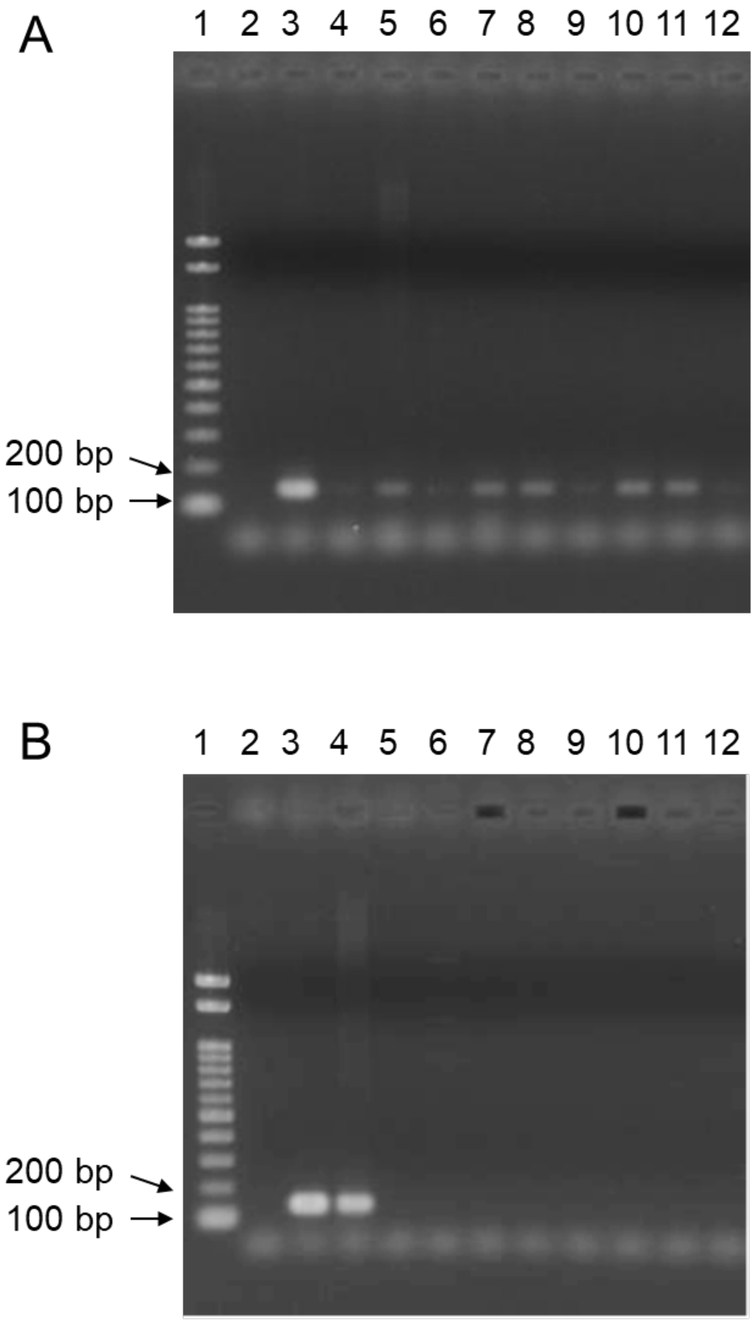

Fig. S3. Agarose gel electrophoreses showing *Rosellinia necatrix* DNA amplified via a species-specific polymerase chain reaction assay. Soil DNA samples were used for as templates after the fungal inoculation accompanied by (A) or not accompanied by (B) sowing of rescue grass (*Bromus catharticus*) in planter boxes (n=3). Lane 1: the size maker (Gene Ladder 100, Nippon gene), Lane 2: the negative control (no template DNA), and Lane 3: the positive control (*R. necatrix* DNA). Lanes 4−6, Lanes 7−9, and Lanes 10−12 contain DNA samples extracted from the soils 3, 25, and 50 cm from the fungal inoculum, respectively.

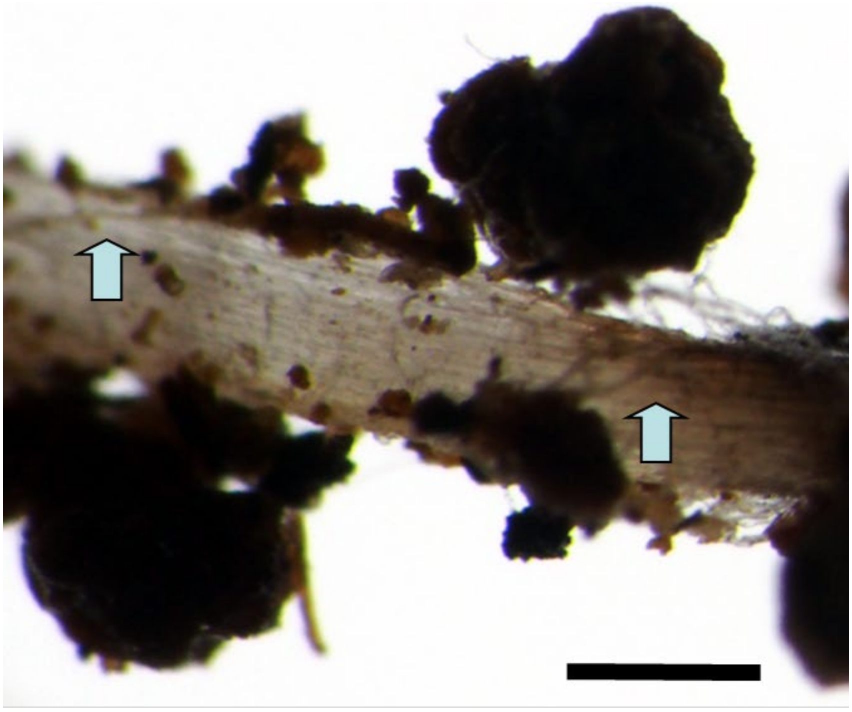

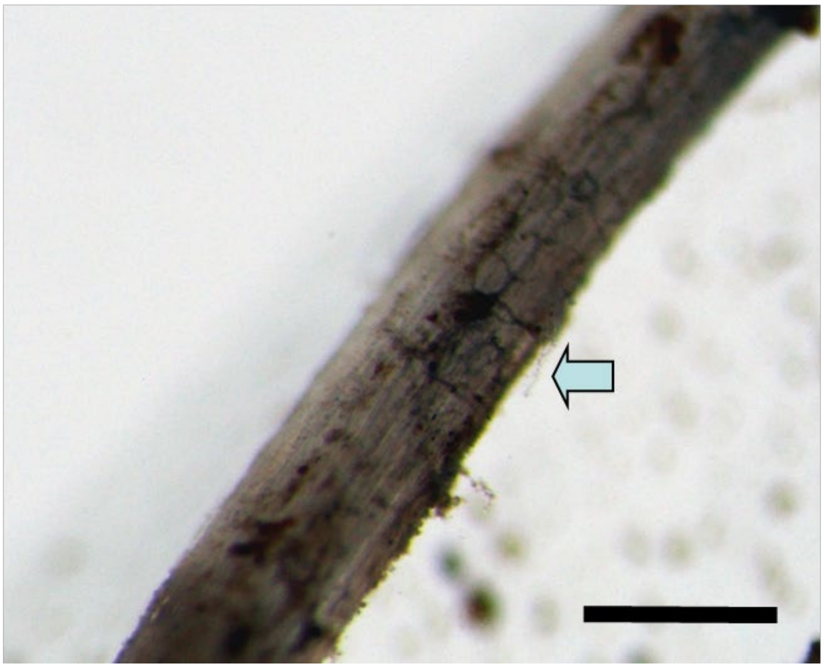

Fig. S4. Mycelial strand-like structures (arrows) observed on the root surface of rescue grass (*Bromus catharticus*) using a dissecting microscope after inoculation of *Rosellinia necatrix*. Bars are 0.3 mm.
